# Validation of the adult attention-deficit/hyperactivity disorder quality-of-life scale in European patients: comparison with patients from the USA

**DOI:** 10.1007/s12402-014-0160-z

**Published:** 2015-01-07

**Authors:** Meryl Brod, Lenard A. Adler, Sarah Lipsius, Yoko Tanaka, Alexandra N. Heinloth, Himanshu Upadhyaya

**Affiliations:** 1 The Brod Group, Mill Valley, CA USA; 2 New York University School of Medicine, New York, NY USA; 3 inVentiv Health Clinical, LLC, Ann Arbor, MI USA; 4 Eli Lilly and Company, Indianapolis, IN USA

**Keywords:** Atomoxetine, Exploratory factor analysis, Convergent validity, Discriminate validity, Responsiveness

## Abstract

The adult attention-deficit/hyperactivity disorder (ADHD) quality-of-life (AAQoL) scale was previously validated in adult patients in the USA; here, the AAQoL is validated in adult European patients. Data from a 12-week open-label acute treatment period with atomoxetine (80–100 mg/day) in adults with ADHD were used. Patients (≥18 to ≤50 years old) had a score ≥2 on ≥6 items on the inattentive or hyperactive core subscales of Conners’ Adult ADHD Rating Scale-Investigator Rated: Screening Version (CAARS-Inv:SV); a CAARS-Inv:SV 18-item total ADHD symptom score ≥20; and Conners’ Adult ADHD Rating Scale-Observer: Screening Version 6-item inattentive or hyperactive core subscale scores ≥2. Data were stratified based on patients’ geographic region (Europe vs USA). Scale validation psychometric properties results were very similar between European (*n* = 1,217; 57.7 % male; mean age 33.0 years) and US (*n* = 602; 62.1 % male; mean age 33.5 years) patients, including factor loading, internal consistency, convergent and discriminant validity, and responsiveness. Exploratory factor analysis confirmed four AAQoL subscales. Internal consistency was acceptable (Cronbach’s alpha > 0.70 for all subscales). The AAQoL total score showed moderate convergent validity with CAARS-Inv:SV 18-item total ADHD symptom and clinical global impression-ADHD-severity (CGI-ADHD-S) scores; and strong convergent validity with Behavior Rating Inventory of Executive Function—Adult Version: Self-Report Global-Executive-Composite Index scores. Mean AAQoL total scores were significantly different among patients grouped by CGI-ADHD-S scores, suggesting good discriminant validity. The AAQoL total and subscale scores presented good responsiveness from baseline to 12 weeks. The AAQoL scale shows comparable validity in European and US adults with ADHD.

**Electronic supplementary material:**

The online version of this article (doi:10.1007/s12402-014-0160-z) contains supplementary material, which is available to authorized users.

## Background

Effects of attention-deficit/hyperactivity disorder (ADHD) in adults go beyond symptoms of inattention, hyperactivity, and impulsivity that characterize the disorder (Adler et al. 2008; Matza et al. 2011). The impact of ADHD involves many aspects of the patient’s life, such as lack of organization, difficulty concentrating, forgetfulness, greater employment disruption, lower academic achievement, difficulty initiating and maintaining relationships, and poor driving behaviors (Adler et al. 2008). It has been shown that ADHD is associated with increased psychological dysfunction and disability, significant job impairment, drug and alcohol misuse, family conflicts, violence, traffic violations, and accidents (Adler et al. 2009). Not surprisingly, patients with ADHD report lower quality-of-life (QoL) than healthy comparison subjects, and the severity of ADHD symptoms is negatively correlated with measures of QoL (Adler et al. 2009; Mattos et al. 2012). Moreover, at least in children with ADHD, the overall impact of the disease is comparable to other major psychiatric disorders or to severe physical conditions (Biederman et al. 2006a; Danckaerts et al. 2010).

The adult ADHD quality-of-life (AAQoL) scale assesses QoL in adult patients with ADHD (Brod et al. 2006). It was developed based on qualitative data on the impact of ADHD on everyday activities as reported by patients and experts, as well as information collected from the scientific literature (Mattos et al. 2011). The AAQoL scale development followed the industry guidance “Patient-Reported Outcome Measures: Use in Medical Product Development to Support Labeling Claims” set by the Food and Drug Administration (http://www.fda.gov/downloads/Drugs/GuidanceComplianceRegulatoryInformation/Guidances/UCM193282.pdf.). In 2006, the AAQoL was first validated in a retrospective cohort study conducted in the USA that included adult patients with ADHD (*n* = 989) treated with atomoxetine. Psychometric validation results showed the ability of the AAQoL to quantify the QoL consequences of ADHD (Brod et al. 2006).

While the AAQoL has been validated and successfully used in adult US patients with ADHD, validation data in European patients are lacking. Here, we examine the validity of the AAQoL in adult European patients with ADHD treated with atomoxetine. We compare our results with data from adult US patients with ADHD treated with atomoxetine who participated in the same clinical trial.

## Methods

This manuscript presents the results of secondary analyses of clinical trial data from an open-label treatment period, focusing on the validation of the AAQoL as a measurement scale in adult European patients with ADHD. The results of the primary study objective, examining the maintenance of response to atomoxetine compared with placebo in adult patients with ADHD, were published elsewhere (Upadhyaya et al. 2013a, b).

### Study design

Data were used from a Phase 3, randomized, double-blind maintenance-of-response trial of atomoxetine (80–100 mg/day) versus placebo in adult outpatients with ADHD. Data were used from the open-label treatment phase of the study, during which all enrolled patients received treatment with atomoxetine (starting dose: 40 mg/day; target dose: 80 or 100 mg/day) for 12 weeks. The study was conducted in 152 centers across 18 countries. For the current analyses, only data collected during the 12-week open-label treatment period in 50 centers in the US and in 82 centers in European countries were used. European countries included Austria, Belgium, Denmark, Finland, France, Germany, Italy, the Netherlands, Portugal, Spain, Sweden, Switzerland, and the UK. Data from additional non-European study centers located in Argentina, Canada, Mexico, and Russia were not included in the present analyses.

### Patients

Adults (≥18 to ≤50 years old) of either gender with a current and historical diagnosis of ADHD, as defined by *Diagnostic and Statistical Manual of Mental Disorders, Fourth Edition, Text Revision™* (DSM-IV-TR™) criteria and a score of ≥2 on at least 6 items of either the inattentive or hyperactive core subscales of the Conners’ Adult ADHD Rating Scale-Investigator Rated: Screening Version (CAARS-Inv:SV) with adult ADHD prompts, were enrolled. In addition, patients had a CAARS-Inv:SV total ADHD symptom score of ≥20, a score of ≥2 on at least 6 items of either the inattentive or hyperactive core subscales of conners’ adult attention-deficit/hyperactivity disorder rating scale-observer: screening version (CAARS-O:SV), and a score of ≥4 on the CGI-ADHD-S. Excluded were patients who met full DSM-IV-TR diagnostic criteria for any history of bipolar disorder, current major depression, a current anxiety disorder (including generalized anxiety disorder, panic disorder, or social phobia), or any history of a psychotic disorder (confirmed by the structured interview); patients with HAMD-17 or HAMA scores of ≥15; and patients with organic brain disease.

### Rating scales

In the current analyses, four different rating scales assessing disease severity were included—the AAQoL, the CAARS-Inv:SV total ADHD symptom scale, the CGI-ADHD-S scale, and the Behavior Rating Inventory of Executive Function—Adult Version: Self-Report (BRIEF-A) scale. All scales were translated using well-recognized scientific guidelines for translation of patient-reported outcome measures (Lohr et al. 1996) and administered in 11 different languages (Danish, Dutch, English, Finnish, French, German, Italian, Portuguese, Russian, Spanish, and Swedish), with the language of the scale depending on the prevalent language(s) of the patients’ country of residence.

The AAQoL is a 29-item questionnaire designed to assess QoL and was a secondary efficacy measure in this trial (access information: www.thebrodgroup.net). It includes four domains (subscale scores): (1) Life Productivity (11 items), including “getting things done on time,” “completing projects or tasks,” “remembering important things,” and “balancing multiple projects;” (2) Psychological Health (6 items), including “feeling anxious,” “overwhelmed,” and “fatigued;” (3) Relationships (5 items), including “tension,” “annoyance,” and “frustration in relationships;” (4) Life Outlook (7 items), including “perceptions that energy is well spent,” “people enjoy spending time with you,” “you can successfully manage your life,” and “you are as productive as you would like to be.” Each item is rated by patients on a 5-point Likert scale ranging from “Not at all/Never” (1) to “Extremely/Very Often” (5). To derive overall and subscale scores, item scores are transformed to a 0–100-point scale. Then, the item scores are summed up and divided by item count to generate subscale and overall scores. If >1 item of a subscore was missing, the subscore was treated as missing. If >3 items for the overall score were missing, the overall score was treated as missing (HCP Team: http://www.hcplive.com/publications/DIALOGS-ADHD/2007/Jun2007/Dialogs_ADHD_Quality_of_Life.). A higher score indicates greater QoL (Brod et al. 2006).

The CAARS-Inv:SV total ADHD symptom scale and the CGI-ADHD-S scale were used as primary efficacy measures in this trial. The CAARS-Inv:SV total ADHD symptom scale consists of the Inattention and Hyperactivity/Impulsivity subscales of the CAARS-Inv:SV. The CAARS-Inv:SV is a 30-item scale containing three subscales: the Inattention subscale (items 1, 9, 13, 14, 19, 21, 26, 29, and 30), the Hyperactivity/Impulsivity subscale (items 2, 4, 6, 8, 16, 18, 22, 25, and 27), and the ADHD Index (items 3, 5, 7, 10, 11, 12, 15, 17, 20, 23, 24, and 28) (Conners et al. 1999). Each item on the CAARS-Inv:SV assesses symptom severity over the previous week and is scored on a 0–3 scale (0 = not at all, never; 1 = just a little, once in a while; 2 = pretty much, often; 3 = very much, very frequently). Adult ADHD prompts were embedded into the 18 items for the total ADHD symptom score (Upadhyaya et al. 2013a). An ADHD symptom was considered to be present if the score on the corresponding item was ≥2. The scale was scored by qualified raters based on interviews with the patients.

The CGI-ADHD-S is a single-item scale. It is rated based on the clinician’s assessment of the overall severity of the patient’s ADHD in relation to the clinician’s total experience (National Institute of Mental Health 1985; Guy 1976). Severity is rated on a 7-point scale (1 = normal or not at all ill; 2 = borderline mentally ill; 3 = mildly ill; 4 = moderately ill; 5 = markedly ill; 6 = severely ill; 7 = extremely ill).

The BRIEF-A was a secondary efficacy measure in this trial. The BRIEF-A is a standardized self-report measure that captures adults’ views of their own executive functions, or self-regulation, in their everyday environments (Roth et al. 2005). It is comprised of 75 equivalent items within nine non-overlapping theoretically and empirically derived clinical scales that measure different aspects of executive functioning: inhibit, shift, emotional control, self-monitor, initiate, working memory, plan/organize, task monitor, and organization of materials. The individual clinical scales form two broader indices: Behavioral Regulation Index and Metacognition Index. These indices form the overall summary score, the Global-Executive-Composite (GEC) Index.

### Statistical analyses

Data from all European and US patients who enrolled in the 12-week open-label treatment phase were included in the analyses. Data were analyzed in three groups: European patient group, US patient group, as well as both groups combined. To analyze the data, statistical software SAS^®^ version 9.1.3 (Cary, NC) was used.

#### Exploratory factor analysis

An exploratory factor analysis was used to examine the underlying constructs of the AAQoL in the European patient group and the US patient group. The number of factors (4) examined in the analysis was based upon the known number of AAQoL subscales, and a value of ≥0.30 was deemed to indicate successful factor loading (Cronbach 1951). Principal-components analysis with varimax rotation was used to estimate the factor loadings and, in turn, determine the underlying factor structure of this study’s AAQoL items in European and US patient groups (Reid 1995).

#### Internal consistency

The degree to which each item of a rating scale co-varies is captured by measures of internal consistency. Internal consistency for the AAQoL total and subscale scores was assessed at baseline and week 12 by use of Cronbach’s alpha (α), a measure of the average correlation of items within a scale (Cronbach 1951). Cronbach’s α ranges from 0 to 1, with higher scores indicating greater internal consistency. A commonly accepted minimal standard for internal consistency is a Cronbach’s α of 0.65 (Zhang et al. 2005). When comparing groups, Cronbach’s α values of 0.70–0.80 are regarded as satisfactory (Bland and Altman 1997).

#### Convergent validity

Convergent validity estimates the degree to which any two measures that assess the same or similar entities are related to each other (Stratford et al. 1996). Convergent validity between AAQoL total score and CAARS-Inv:SV total ADHD symptom score, CGI-ADHD-S score, and BRIEF-A GEC Index score was assessed at week 12 using Pearson correlation coefficients. In an exploratory analysis, Pearson correlation coefficients of AAQoL subscale scores versus AAQoL total scores, CAARS-Inv:SV, hyperactive/impulsive and inattentive scores, CGI-ADHD-S score, and BRIEF-A Metacognition, Behavioral Regulation, and GEC Index scores at week 12 were determined. Week 12 was the last non-missing value during the 12-week open-label acute treatment period.

Convergent validity was assessed with Pearson correlation coefficients. In the current analyses, correlations with a coefficient value ≤0.5 were classified as weak; those with a correlation coefficient of >0.5 but <0.8 as moderate; and those with a correlation coefficient of ≥0.8 as strong.

#### Discriminant validity

Discriminant validity indicates the ability to discriminate between dissimilar constructs (Stratford et al. 1996)—here, the ability of a scale to discriminate between patient groups with differences in their QoL was assessed. A measure for QoL in patients with ADHD should distinguish between patients with different levels of QoL. Here, comparisons of AAQoL total scores between patients grouped by CGI-ADHD-S scores at 12 weeks, indicating severity of the disease, were performed. Mean AAQoL total scores were compared between patients with CGI-ADHD-S scores of 1 (normal) versus patients with CGI-ADHD-S scores of 2 through 5 (borderline mentally ill up to markedly ill; 5 = highest CGI-ADHD-S score at week 12 with a sufficient number of affected patients to produce statistically meaningful results). An analysis of variance was conducted using AAQoL total score as the outcome and CGI-ADHD-S as the predictor; *P* values were obtained by pairwise comparison.

#### Responsiveness

Responsiveness is the extent to which a health status measure accurately reflects change in a patient’s condition over time (Matza et al. 2007). The standardized response mean (SRM) is a unitless statistic summarizing responsiveness, defined as the mean change from baseline score divided by the standard deviation of the change scores (Biederman et al. 2006b), similar in concept to effect sizes, but using only data from one treatment group. Wilcoxon signed-rank tests were used to assess within-group changes from baseline to week 12 for the AAQoL total and subscale scores based on clinical assessment of improvement as measured by the CGI-ADHD-S.

## Results

### Patient demographics

A total of 1,819 adult patients with ADHD were considered for this analysis. Among them, 1,217 patients resided in European countries and 602 patients lived in the US. Baseline demographic and clinical characteristics are presented in Table 1. Further comparisons of baseline patient characteristics between European and non-European patients were published previously (Upadhyaya et al. 2013a).

**Table 1:** Baseline demographics

Characteristic	EC (*N* = 1,217)	US (*N* = 602)	EC + US (*N* = 1,819)	*P* value
Age [years, mean (SD)]	33.0 (9.2)	33.5 (8.9)	33.2 (9.1)	0.29^a^
*Race [n (%)]*				<0.001^b^
Caucasian	1,192 (97.9)	486 (80.7)	1,678 (92.2)	
African	5 (0.4)	50 (8.3)	55 (3.0)	
Hispanic	9 (0.7)	44 (7.3)	53 (2.9)	
Native American	1 (0.1)	5 (0.8)	6 (0.3)	
East Asian	3 (0.2)	11 (1.8)	14 (0.8)	
West Asian (Indian subcontinent)	7 (0.6)	5 (0.8)	12 (0.7)	
Not provided	0 (0.0)	1 (0.2)	1 (0.1)	
*Gender [n (%)]*				0.08^b^
Male	702 (57.7)	374 (62.1)	1,076 (59.2)	
Female	515 (42.3)	228 (37.9)	743 (40.8)	
Body weight [kg, mean (SD)]	76.8 (17.6)	84.1 (19.3)	79.2 (18.5)	<0.001^a^
Height [cm, mean (SD)]	173.8 (9.3)	172.3 (10.0)	173.3 (9.6)	0.002^a^
BMI [kg/m^2^, mean (SD)]	25.3 (5.0)	28.3 (5.9)	26.3 (5.5)	<0.001^a^
*ADHD subtype [n (%)]*				<0.001^b^
Inattentive	298 (24.5)	115 (19.1)	413 (22.7)	
Hyperactive/impulsive	40 (3.3)	4 (0.7)	44 (2.4)	
Combined	872 (71.7)	482 (80.1)	1,354 (74.4)	
Not applicable	1 (0.1)	0 (0.0)	1 (0.1)	
CGI-ADHD-S score [mean (SD)]	5.1 (0.8)	4.6 (0.6)	5.0 (0.8)	<0.001^a^
*CAARS*-*Inv:SV score [mean (SD)]*				
Hyperactivity–Impulsivity subscale imputed	17.4 (5.2)	18.3 (4.9)	17.7 (5.1)	<0.001^a^
Inattention subscale imputed	20.9 (3.6)	21.6 (3.6)	21.1 (3.6)	<0.001^a^
Total ADHD symptom imputed	38.3(6.7)	39.9(6.8)	38.8(6.8)	<0.001^a^
*AAQoL [mean (SD)]*				
Total score imputed	47.6 (14.3)	48.0 (14.0)	47.7 (14.2)	0.53^a^
Psychological Health section score	51.4 (20.9)	53.1 (19.2)	52.0 (20.3)	0.09^a^
Relationships section score	53.6 (21.2)	52.8 (19.1)	53.3 (20.5)	0.44^a^
Life Outlook section score	45.1 (17.0)	52.5 (14.9)	47.6 (16.7)	<0.001^a^
Life Productivity section score	44.2 (18.1)	40.3 (19.0)	42.9 (18.5)	<0.001^a^

European countries include: Austria, Belgium, Denmark, Finland, France, Germany, Italy, the Netherlands, Portugal, Spain, Sweden, Switzerland, and the UK

*AAQoL* adult attention-deficit/hyperactivity disorder quality-of-life, *ADHD* attention-deficit/hyperactivity disorder, *BMI* body mass index; *CAARS*-*Inv:SV* conner’s adult attention-deficit/hyperactivity disorder investigator rated: screening version, *CGI*-*ADHD*-*S* clinical global impression attention-deficit/hyperactivity disorder-severity, *EC* European countries, *N* total number of patients, *n* number of affected patients, *SD* standard deviation, *US* United States

^a^ *P* values are from *t* test

^b^ *P* values are from Fisher’s exact test

### Exploratory factor analysis

Exploratory factor analysis loaded all AAQoL items onto their previously reported subscales (Brod et al. 2006) with the exception of item #29 (your intimate relationship is going well emotionally), which loaded on the Relationships subscale instead of the Life Outlook subscale. Loading and significance of loading were very similar between European and US patients for all items including item #29 (Table 2).

**Table 2:** Exploratory factor analysis for adult attention-deficit/hyperactivity disorder quality-of-life scale

Subscale	Item	Item description	Factor loading		
			EC *n* = 1,137	US *n* = 594	EC + US *n* = 1,731
Life productivity	1	Keep the house/apartment clean or uncluttered	0.620	0.651	0.627
	2	Manage your finances (such as cashing checks, balancing your checkbook, paying bills on time)	0.553	0.596	0.582
	3	Remember important things	0.562	0.686	0.624
	4	Get your shopping done (such as for food, clothes, or household items)	0.514	0.621	0.576
	5	Pay attention when interacting with others	0.417	0.588	0.507
	11	Getting things done requires too much effort	0.569	0.613	0.554
	22	Complete projects or tasks (either at work or at home)	0.758	0.721	0.739
	23	Get started with tasks you do not find interesting	0.686	0.685	0.678
	24	Balance multiple projects	0.647	0.672	0.661
	25	Get things done on time	0.764	0.719	0.733
	26	Keep track of important items (such as keys, wallet)	0.502	0.573	0.553
Psychological health	6	Overwhelmed	0.463	0.634	0.518
	7	Anxious	0.725	0.741	0.731
	8	Depressed	0.700	0.728	0.721
	13	You have overreacted in difficult or stressful situations	0.394	0.321	0.421
	20	Feeling fatigued	0.603	0.514	0.584
	21	Fluctuations (ups and downs) in your emotions	0.671	0.674	0.703
Life outlook	14	Your energy is well spent (has positive results)	0.703	0.703	0.713
	15	Able to enjoy time spent with others	0.644	0.550	0.618
	16	You can successfully manage your life	0.700	0.655	0.693
	17	As productive as you would like to be	0.683	0.646	0.664
	27	Good about yourself	0.666	0.633	0.678
	28	People enjoy spending time with you	0.521	0.597	0.541
	29^a^	Your intimate relationship is going well emotionally	0.155	0.018	0.091
Relationships	9	You have not been able to meet others’ expectations of you (either at home or at work)	0.364	0.305	0.333
	10	You annoyed people	0.731	0.662	0.716
	12	People are frustrated with you	0.710	0.637	0.697
	18	Tension in relationships	0.556	0.569	0.515
	19	Not having quality time to spend with others	0.391	0.566	0.394
	29^a^	Your intimate relationship is going well emotionally	−0.306	−0.268	−0.306

European countries include Austria, Belgium, Denmark, Finland, France, Germany, Italy, the Netherlands, Portugal, Spain, Sweden, Switzerland, and the UK

*AAQoL* Adult attention-deficit/hyperactivity disorder quality-of-life, *EC* European countries, *n* number of patients with a baseline value, *US* United States

^a^Item #29 loaded in our study for European and US groups on the AAQoL Relationships subscale instead of AAQoL Life Outlook subscale. This may be due to the fact that conceptually the item could belong to both Life Outlook and Relationships subscales

### Internal consistency

Cronbach’s α, a measure of internal consistency, at baseline, was 0.744 for AAQoL total scores in European patients and 0.771 in US patients; at week 12, Cronbach’s α was 0.835 in European patients and 0.851 in US patients. The internal consistency score improved from baseline to week 12 in response to treatment with atomoxetine in both European and US patients. For all four AAQoL subscales (Life Productivity, Psychological Health, Life Outlook, and Relationships), Cronbach’s α values were >0.70 at baseline and week 12, indicating acceptable internal consistency in European and US patients.

Overall, very similar values for Cronbach’s α were observed for European and US patients. Internal consistency remained high and acceptable with Cronbach’s α > 0.70 when patients from European and US groups were combined, at both baseline and week 12 (Table 3).

**Table 3:** Internal consistency as represented by AAQoL subscales: Cronbach’s Alpha at baseline and week 12

Variable category	EC		US		EC + US	
	Baseline	Week 12	Baseline	Week 12	Baseline	Week 12
Life productivity subscale	0.854 *n* = 1,180	0.920 *n* = 1,023	0.881 *n* = 600	0.929 *n* = 491	0.864 *n* = 1,780	0.923 *n* = 1,514
Psychological health subscale	0.772 *n* = 1,188	0.841 *n* = 1,030	0.778 *n* = 601	0.843 *n* = 492	0.766 *n* = 1,789	0.839 *n* = 1,522
Life outlook subscale	0.786 *n* = 979	0.846 *n* = 876	0.781 *n* = 501	0.853 *n* = 403	0.791 *n* = 1,480	0.854 *n* = 1,279
Relationships subscale	0.751 *n* = 1,190	0.775 *n* = 1,028	0.754 *n* = 601	0.790 *n* = 492	0.752 *n* = 1,791	0.779 *n* = 1,520
Total score	0.893 *n* = 950	0.939 *n* = 852	0.901 *n* = 498	0.946 *n* = 399	0.894 *n* = 1,448	0.941 *n* = 1,251

European countries include: Austria, Belgium, Denmark, Finland, France, Germany, Italy, the Netherlands, Portugal, Spain, Sweden, Switzerland, and the UK

*AAQoL* adult attention-deficit/hyperactivity disorder quality-of-life, *EC* European countries, *n* number of patients, *US* United States

### Convergent validity

As assessed with Pearson correlation coefficients, at 12 weeks, AAQoL total scores demonstrated moderate convergent validity with CAARS-Inv:SV total ADHD symptom and CGI-ADHD-S scores, in both European and US patient populations. Correlations between AAQoL total scores and BRIEF-A GEC Index scores were strong in both European and US patient populations (Supplemental Table 1).

Overall, European and US patient populations showed very similar correlation values between AAQoL total scores and CAARS-Inv:SV total ADHD symptom scores, CGI-ADHD-S scores, and BRIEF-A GEC Index scores, indicating very similar convergent validity in European and US patient groups (Supplemental Table 1).

In an exploratory analysis, Pearson correlation coefficients between AAQoL subscale scores and AAQoL total scores as well as CAARS-Inv:SV hyperactive/impulsive score, CAARS-Inv:SV inattentive score, CGI-ADHD-S score, BRIEF-A Metacognition Index score, BRIEF-A Behavioral Regulation Index score, and BRIEF-A GEC Index score at 12 weeks were assessed (Supplemental Table 1).

All four AAQoL subscale scores had moderate-to-strong correlations with the AAQoL total score in European and US patients. Correlations with the CAARS-Inv:SV hyperactive/impulsive subscale score were weak for all AAQoL subscale scores in European and US patient groups. For the CAARS-Inv:SV inattentive subscale, correlations with AAQoL subscale scores were weak to moderate for European and US patient groups. All AAQoL subscale scores showed weak correlations with the CGI-ADHD-S score with the exception of the Life Productivity subscale, which demonstrated moderate correlation with the CGI-ADHD-S score in both European and US patients. The AAQoL Productivity subscale score was also moderately correlated with both the BRIEF-A Metacognition Index score and the BRIEF-A GEC Index score. For the remaining 3 AAQoL subscales, scores were low moderately correlated with the BRIEF-A Metacognition Index score and the BRIEF-A GEC Index score. Correlations between all AAQoL subscale scores and the BRIEF-A Behavioral Regulation Index score were low-moderate to moderate in European and US patient groups (Supplemental Table 1). Overall, correlations between AAQoL subscale scores and comparator scale scores were very similar in European and US patient groups.

### Discriminant validity

**Fig. 1:**
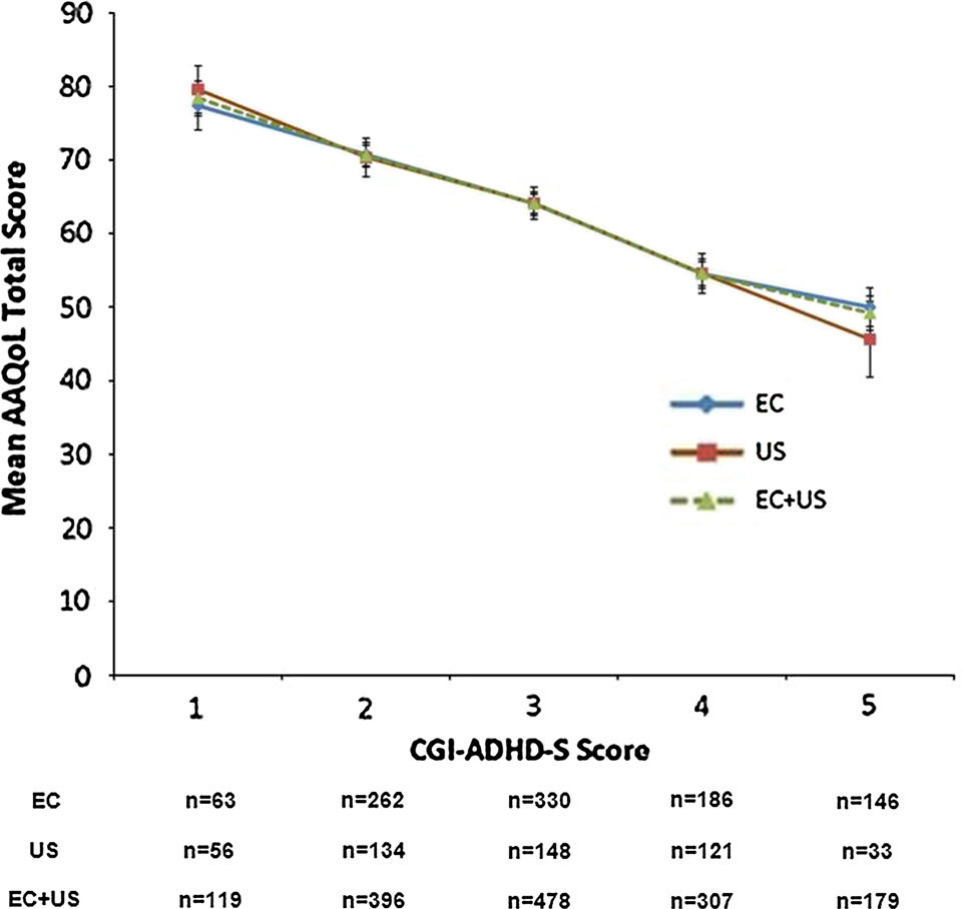
Comparison of AAQoL total scores and CGI-ADHD-S scores at week 12. *AAQoL* adult attention-deficit/hyperactivity disorder quality-of-life, *CGI*-*ADHD*-*S* clinical global impression attention-deficit/hyperactivity disorder-severity, *EC* European countries, *n* number of subjects, *US* United States

Overall, discriminant validity (measured with analysis of variance) was very similar between European and US groups, with comparable mean AAQoL scores for patient groups with identical CGI-ADHD-S scores. Mean AAQoL total scores decreased with increasing CGI-ADHD-S scores in European and US patients, indicating lower QoL in patients with higher ADHD symptom severity. Furthermore, analyses revealed significant (*P* ≤ 0.0001 for European and US patient groups) differences in mean AAQoL total scores at week 12 between patients with a CGI-ADHD-S score of 1 versus patients with CGI-ADHD-S scores of 2 through 5, indicating discriminant validity of the AAQoL (Fig. 1; too few patients had CGI-ADHD-S scores >5 at week 12 for statistically meaningful analyses).

### Responsiveness

The AAQoL total and subscale scores showed significant (Wilcoxon signed-rank tests, *P* < 0.001) mean changes from baseline to week 12 in European and US patient groups, indicating good responsiveness.

Mean AAQoL total and subscale score changes were similar between European and US patient groups. Additionally, SRMs were comparable between European and US patient groups (Table 4), indicating similar responsiveness in European and US patient groups.

**Table 4:** Responsiveness analysis: mean change from baseline to week 12

AAQoL	EC				US				EC + US			
	*n*	Baseline mean (SD)	Mean change at week 12 (SD)	SRM	*n*	Baseline mean (SD)	Mean change at week 12 (SD)	SRM	*n*	Baseline mean (SD)	Mean change at week 12 (SD)	SRM
Life productivity subscale	1,026	44.1 (18.12)	19.9 (22.07)^a^	0.902	492	40.7 (19.13)	21.9 (24.35)^a^	0.899	1,518	43.0 (18.51)	20.6 (22.85)^a^	0.902
Psychological health subscale	1,028	51.4 (20.90)	12.4 (22.29)^a^	0.556	493	53.0 (18.78)	12.2 (21.56)^a^	0.566	1,521	51.9 (20.24)	12.4 (22.05)^a^	0.562
Life outlook subscale	986	45.2 (16.98)	8.5 (19.74)^a^	0.431	488	52.1 (14.66)	10.4 (17.91)^a^	0.581	1,474	47.4 (16.57)	9.1 (19.17)^a^	0.475
Relationships subscale	1,029	53.8 (21.31)	12.7 (23.04)^a^	0.551	493	53.1 (19.29)	14.2 (22.83)^a^	0.622	1,522	53.6 (20.68)	13.2 (22.98)^a^	0.574
Total score	1,025	47.6 (14.31)	14.4 (17.71)^a^	0.813	493	48.1 (14.01)	15.8 (18.19)^a^	0.869	1,518	47.7 (14.21)	14.9 (17.87)^a^	0.834

European countries include: Austria, Belgium, Denmark, Finland, France, Germany, Italy, the Netherlands, Portugal, Spain, Sweden, Switzerland, and the UK

*AAQoL* adult attention-deficit/hyperactivity disorder quality-of-life, *EC* European countries, *n* number of patients, *SD* standard deviation, *SRM* standardized response mean, *US* United States

^a^Statistically significant (*P* < 0.001) mean change from baseline to week 12 based on Wilcoxon signed-rank test

## Discussion

The analyses presented here provide evidence that the AAQoL is a valid measure of ADHD-related QoL in adult European patients. While the AAQoL has previously been validated in US patients, this is the first validation of the AAQoL in European patients. Although this clinical trial was not designed to test the validity of the AAQoL, it allowed a comparative validation of the scale in one subpopulation (European patients) versus another subpopulation (US patients) in which the AAQoL had been previously validated. Overall, results of all measures for internal consistency, convergent and discriminant validity, and responsiveness were very similar between regions within our study, as well as between our study populations and a prior validation of the AAQoL (Brod et al. 2006).

The exploratory factor analysis confirmed the valid discrimination of four AAQoL subscales in European and US patient groups, with similar loading values for all items in European and US patients. With the exception of item #29 (your intimate relationship is going well emotionally), the previously published (Brod et al. 2006) factor structure was confirmed in European and US patients. Item #29 loaded for European and US patients on the AAQoL Relationships subscale; previously, it has been reported to belong to the AAQoL Life Outlook subscale. While it is not surprising that item #29 originally loaded on the Life Outlook subscale, as it refers to a positive perspective in the patient’s life, it is also not surprising that it could load on the Relationships subscale, as it specifically refers to the quality of the patient’s relationship. Conceptually, item #29 could be in either Life Outlook or Relationships subscales.

The AAQoL demonstrated acceptable internal consistency at both baseline and week 12 with Cronbach’s α values of >0.70 for total and subscale scores in European and US patient populations, which is consistent with prior findings (Brod et al. 2006).

Convergent validity of the AAQoL total score at week 12 was weak to moderate with the CAARS-Inv:SV total ADHD symptom score and the CGI-ADHD-S score and was moderate to strong with the BRIEF-A GEC Index score. These findings are consistent with the fact that the AAQoL was created to specifically address the impact of ADHD symptoms on the life of patients, and some of its questions overlap with questions targeting ADHD symptoms or impairment caused by ADHD in the patients. The moderate-to-strong correlation between the AAQoL and the BRIEF-A GEC Index is also not surprising, as deficits in executive functioning have been demonstrated to be an essential abnormality in ADHD (Coghill 2010). Moreover, it is expected that deficits in executive functioning, which are impairments that the individual has on planning, organizing, and executing practical tasks in life would be associated with a worse perception of QoL.

As expected, all correlation values between AAQoL and comparator scale scores were negative due to the scale definitions: A higher score on the AAQoL indicates better life functioning, while lower scores on the comparator scales indicate a decreased presence of symptoms.

Discriminant validity was assessed by measuring the ability of the AAQoL to discriminate among patients grouped by their week 12 CGI-ADHD-S scores, because no scale that would be suitable for the assessment of discriminant validity was included in the clinical trial. Therefore, we chose to address discriminant validity as the ability of the AAQoL to discriminate between different levels of ADHD severity. For European and US patients, mean AAQoL scores were significantly different between patients with a CGI-ADHD-S score of 1, indicating mentally normal, versus patients with CGI-ADHD-S scores of 2–5, indicating borderline mentally ill up to markedly ill. This suggests that the AAQoL was able to discriminate groups of patients with different disease severity. Overall, results for discriminant validity were very similar between European and US patient groups.

These findings suggest that the AAQoL is a valuable tool to assess treatment effects in clinical trials, addressing an important need in the field. Within the ADHD research community, an increasing demand to incorporate assessments of treatment effects that go beyond pure symptomatic amelioration is being recognized (Coghill 2010). Poor QoL has been identified as an important dimension to be evaluated when assessing treatment effects in clinical trials. The similar responsiveness in European and US patient groups suggests that the AAQoL is an adequate tool for evaluating treatment effects in both geographic regions. While the tool is primarily used in clinical trials, it might also be useful for clinical practitioners when assessing treatment success in their adult patients.

Overall, the moderate correlation between the AAQOL and the CAARS-Inv:SV supports the idea that the 18-item total ADHD symptom score listed in the *Diagnostic and Statistical Manual of Mental Disorders* (DSM) does not capture the full impact of ADHD on QoL. Consequently, QoL assessment at baseline and during treatment is of much importance. Both DSM symptoms and QoL should be assessed to demonstrate improvement in patients with ADHD, as expressed in the European Medical Agency (EMA) guidelines for drug development in ADHD.

The interpretation of the results of the current study is limited by the open-label study design. Because this study was not designed for a priori investigation of psychometric properties of the AAQoL in a European population, the scales that were chosen as comparators for convergent and discriminant validity were not the standard scales that could be used for that purpose. However, psychometric findings in European and US patient populations in this study were overall very consistent. Together with the prior validation study of the AAQoL in a US population (Brod et al. 2006), the current results suggest that the AAQoL can be used as an adequate measure of QoL in European and US patients. Strengths of the study are the use of a patient population which well represents diverse European regions and the inclusion of a US population in which the AAQoL has previously been validated.

## Conclusions

The AAQoL shows comparable validity in European and US patients, ≥18 to ≤50 years old, for assessing baseline and changes in QoL in adults with ADHD during treatment with atomoxetine. Based on our results, the AAQoL can be a valuable tool to investigate QoL in European adult patients with ADHD and can be used to measure changes in the QoL with treatment in these patients.

## Electronic supplementary material

Below is the link to the electronic supplementary material.

Supplementary material 1 (DOCX 21 kb)
