# Supplementary material for: Validation of the adult attention-deficit/hyperactivity disorder quality-of-life scale in European patients: comparison with patients from the USA
Source: Atten Defic Hyperact Disord. 2015 Jan 7;7(2):141–50. doi: 10.1007/s12402-014-0160-z (PMC4449381; doi:10.1007/s12402-014-0160-z)
Supplement: Supplementary file 1 — Supplementary material 1 (DOCX 21 kb) [file 12402_2014_160_MOESM1_ESM.docx]

**Supplementary Material**

**Supplemental Table 1: Convergent Validity**

| **Correlation between AAQoL Subscale Score** | | **Life Productivity** | **Psychological Health** | **Life Outlook** | **Relationships** | **AAQoL Total Score** |
| --- | --- | --- | --- | --- | --- | --- |
| **Scale** | **Countries** | **Week 12** | **Week 12** | **Week 12** | **Week 12** | **Week 12** |
| AAQoL total score | EC | 0.89*  (0.88,0.90)  n=1034 | 0.84*  (0.82,0.86)  n=1035 | 0.72*  (0.69,0.75)  n=1011 | 0.80*  (0.77,0.82)  n=1034 | - |
|  | US | 0.89*  (0.87,0.91)  n=492 | 0.84*  (0.81,0.86)  n=493 | 0.74*  (0.70,0.78)  n=492 | 0.82*  (0.78,0.84)  n=493 | - |
|  | Total | 0.89*  (0.87,0.90)  n=1526 | 0.84*  (0.82,0.85)  n=1528 | 0.72*  (0.70,0.75)  n=1503 | 0.80*  (0.78,0.82)  n=1527 | - |
| CAARS-Inv:SV total ADHD symptom imputed score | EC | - | - | - | - | -0.57*  (-0.61, -0.52)  n=1033 |
|  | US | - | - | - | - | -0.60*  (-0.65, -0.54)  n=493 |
|  | Total | - | - | - | - | -0.58*  (-0.61, -0.54)  n=1526 |
| CAARS-Inv:SV Hyperactive/Impulsive imputed score | EC | -0.44*  (-0.49,-0.39)  n=1033 | -0.40*  (-0.45,-0.34)  n=1037 | -0.25*  (-0.30,-0.19)  n=1009 | -0.35*  (-0.41,-0.30)  n=1037 |  |
|  | US | -0.49*  (-0.56,-0.42)  n=492 | -0.39*  (-0.46,-0.31)  n=493 | -0.23*  (-0.31,-0.15)  n=492 | -0.42*  (-0.49,-0.34)  n=493 |  |
|  | Total | -0.46*  (-0.50,-0.42)  n=1525 | -0.39*  (-0.44,-0.35)  n=1530 | -0.23*  (-0.28,-0.19)  n=1501 | -0.37*  (-0.42,-0.33)  n=1530 |  |
| CAARS-Inv:SV Inattentive imputed score | EC | -0.63*  (-0.66,-0.59)  n=1033 | -0.41*  (-0.46,-0.36)  n=1037 | -0.36*  (-0.41,-0.31)  n=1009 | -0.37*  (-0.43,-0.32)  n=1037 | - |
|  | US | -0.67*  (-0.72,-0.62)  n=492 | -0.45*  (-0.52,-0.38)  n=493 | -0.36*  (-0.43,-0.28)  n=492 | -0.44*  (-0.51,-0.37  n=493 | - |
|  | Total | -0.64*  (-0.67,-0.61)  n=1525 | -0.42*  (-0.46,-0.38)  n=1530 | -0.35*  (-0.40,-0.31)  n=1501 | -0.40*  (-0.44,-0.35)  n=1530 | - |
| CGI-ADHD-S score | EC | -0.54*  (-0.58,-0.50)  n=1032 | -0.39*  (-0.44,-0.34)  n=1036 | -0.34*  (-0.39,-0.28)  n=1008 | -0.35*  (-0.40,-0.30)  n=1036 | -0.52*  (-0.57,-0.48)  n=1032 |
|  | US | -0.56*  (-0.62,-0.49)  n=492 | -0.40*  (-0.47,-0.32)  n=493 | -0.32*  (-0.39,-0.23)  n=492 | -0.38*  (-0.45,-0.30)  n=493 | -0.53*  (-0.59,-0.47)  n=493 |
|  | Total | -0.54*  (-0.57,-0.50)  n=1524 | -0.39*  (-0.44,-0.35)  n=1529 | -0.35*  (-0.39,-0.31)  n=1500 | -0.36*  (-0.40,-0.31)  n=1529 | -0.53*  (-0.56,-0.49)  n=1525 |
| BRIEF-A Self Raw Metacognition Index score | EC | -0.82*  (-0.84,-0.79)  n=989 | -0.54*  (-0.58,-0.49)  n=993 | -0.45*  (-0.50,-0.40)  n=967 | -0.53*  (-0.57,-0.48)  n=994 | - |
|  | US | -0.76*  (-0.80,-0.72)  n=489 | -0.54*  (-0.60,-0.48)  n=490 | -0.50*  (-0.56,-0.43)  n=489 | -0.57*  (-0.63,-0.51)  n=490 | - |
|  | Total | -0.80*  (-0.81,-0.78)  n=1478 | -0.54*  (-0.58,-0.51)  n=1483 | -0.46*  (-0.50,-0.42)  n=1456 | -0.54*  (-0.58,-0.51)  n=1484 | - |
| BRIEF-A Self Raw Behavioral Regulation Index score | EC | -0.62*  (-0.66,-0.58)  n=990 | -0.67*  (-0.70,-0.63)  n=994 | -0.41*  (-0.46,-0.36)  n=969 | -0.61*  (-0.65,-0.57)  n=995 | - |
|  | US | -0.59*  (-0.65,-0.53)  n=487 | -0.67*  (-0.71,-0.62)  n=488 | -0.46*  (-0.53,-0.39)  n=487 | -0.66*  (-0.71,-0.60)  n=488 | - |
|  | Total | -0.60*  (-0.63,-0.56)  n=1477 | -0.66*  (-0.69,-0.63)  n=1482 | -0.44*  (-0.48,-0.40)  n=1456 | -0.62*  (-0.65,-0.59)  n=1483 | - |
| BRIEF-A Self Raw GEC Index score | EC | -0.80*  (-0.82,-0.77)  n=963 | -0.64*  (-0.68,-0.60)  n=967 | -0.46*  (-0.51,-0.41)  n=944 | -0.61*  (-0.65,-0.57)  n=968 | -0.80*  (-0.82,-0.77)  n=963 |
|  | US | -0.74*  (-0.78,-0.70)  n=485 | -0.63*  (-0.68,-0.58)  n=486 | -0.52*  (-0.58,-0.45)  n=485 | -0.65*  (-0.70,-0.60)  n=486 | -0.79*  (-0.82,-0.75)  n=486 |
|  | Total | -0.77*  (-0.79,-0.75)  n=1448 | -0.64*  (-0.67,-0.61)  n=1453 | -0.49*  (-0.52,-0.44)  n=1429 | -0.62*  (-0.65,-0.59)  n=1454 | -0.79*  (-0.81,-0.77)  n=1449 |

*Significantly different from 0 (*P*≤0.05). Higher scores on the AAQoL indicate better life functioning while lower scores on the comparator scales indicate lower presence of symptoms.

Abbreviations: AAQoL=Adult Attention-Deficit/Hyperactivity Disorder Quality-of-Life; ADHD = adult attention-deficit/hyperactivity disorder; BRIEF-A=Behavior Rating Inventory of Executive Function–Adult Version: Self Report; CAARS-Inv:SV=Conners' Adult Attention-Deficit/Hyperactivity Disorder Investigator Rated: Screening Version; CGI-ADHD-S=Clinical Global Impression-ADHD-Severity; EC=European countries; GEC=Global Executive Composite; n=number of patients; US=United States.

Note: European countries include: Austria, Belgium, Denmark, Finland, France, Germany, Italy, the Netherlands, Portugal, Spain, Sweden, Switzerland, and the United Kingdom.
